# Centralized red muscle in *Odontaspis ferox* and the prevalence of regional endothermy in sharks

**DOI:** 10.1098/rsbl.2023.0331

**Published:** 2023-11-08

**Authors:** Haley R. Dolton, Edward P. Snelling, Robert Deaville, Andrew L. Jackson, Matthew W. Perkins, Jenny R. Bortoluzzi, Kevin Purves, David J. Curnick, Catalina Pimiento, Nicholas L. Payne

**Affiliations:** ^1^ Discipline of Zoology, School of Natural Sciences, Trinity College Dublin, Dublin 2, Ireland; ^2^ Department of Anatomy and Physiology, and Centre for Veterinary Wildlife Research, Faculty of Veterinary Science, University of Pretoria, Pretoria, Gauteng 0110, South Africa; ^3^ Institute of Zoology, Zoological Society of London, Regent's Park, London NW1 4RY, UK; ^4^ School of Veterinary Science, University College Dublin, Belfield, Dublin 4, Ireland; ^5^ Department of Paleontology, University of Zurich, Zurich, Switzerland; ^6^ Department of Biosciences, Swansea University, Swansea, UK; ^7^ Smithsonian Tropical Research Institute, Balboa, Panama

**Keywords:** endothermy, sharks, mesotherm, energetics, megalodon

## Abstract

The order Lamniformes contains charismatic species such as the white shark *Carcharodon carcharias* and extinct megatooth shark *Otodus megalodon*, and is of particular interest given their influence on marine ecosystems, and because some members exhibit regional endothermy. However, there remains significant debate surrounding the prevalence and evolutionary origin of regional endothermy in the order, and therefore the development of phenomena such as gigantism and filter-feeding in sharks generally. Here we show a basal lamniform shark, the smalltooth sand tiger shark *Odontaspis ferox*, has centralized skeletal red muscle and a thick compact-walled ventricle; anatomical features generally consistent with regionally endothermy. This result, together with the recent discovery of probable red muscle endothermy in filter feeding basking sharks *Cetorhinus maximus*, suggests that this thermophysiology is more prevalent in the Lamniformes than previously thought, which in turn has implications for understanding the evolution of regional endothermy, gigantism, and extinction risk of warm-bodied shark species both past and present.

## 1. Introduction

While at least 99% of fish species are ectotherms [1], regional endothermy is a remarkable example of convergent evolution seen in several lineages of large-bodied fish taxa. Several tunas and several families of sharks have evolved a suite of traits such as centralized red muscle, a high percentage of compact myocardium of the ventricle, and counter-current vascular heat exchangers that enable the maintenance of elevated temperature of key tissues above that of ambient water [2]. Various forms of regional endothermy (namely red muscle, cranial, orbital and visceral) are thought to facilitate competitive advantages in apex ‘high performance’ fishes, such as faster cruising speeds, longer migration distances, enhanced visual perception, niche expansion and rapid digestion rates [1,3–6]. The maintenance of elevated temperature within key tissues is an evolutionary triumph over the convective and conductive avenues of heat transfer that would otherwise transfer heat from the body to cooler ambient water [7]. This is especially impressive considering all blood is circulated through the gills and thus comes in close apposition with the water.

In sharks, all known regional endotherms are found within the order Lamniformes [2]. Of the 15 extant Lamniformes, six species have centralized red muscle which is warmer than ambient water (in basking sharks *Cetorhinus maximus* the subcutaneous white muscle is warmer, and red muscle is assumed, but not confirmed, to be warmer; [8]), whereas of the remaining nine species, two have lateral red muscle that is not warmer but they likely exhibit cranial endothermy [9], and seven are untested or commonly assumed to be ectotherms [10] (see electronic supplementary material, table S1). Recently, the thermophysiology and evolutionary history of Lamniformes has received significant attention given ongoing uncertainty on the origins of regional endothermy and associated consequences for the development of gigantism, filter-feeding, and extinction drivers of enigmatic species. For example, the extinct megatooth shark, *Otodus megalodon*, was a 15–20 m (total length (TL)) [11,12] macropredator which undoubtedly held a high trophic position [13] during the Miocene to early Pliocene [14]. The true phylogeny of Lamniformes remains debated, and whether or not *O. megalodon* had regional endothermy is the focus of several recent papers [12,14–16], likely due to its gigantic size and influence on the evolution and ecology of marine ecosystems [14]. Several lines of evidence have been used to infer that *O. megalodon* likely exhibited some form of regional endothermy (e.g. isotopic analysis, inferred swim speeds and energy budget estimation), but also that the high whole-body metabolic demands of being a gigantic, regionally endothermic macropredator contributed to its extinction [10,17–19]. However, an extant massive filter-feeding lamniform, *C. maximus*, was recently shown to be the largest species to date to exhibit regionally endothermic features, with centralized red muscle and sustained elevated subcutaneous white muscle temperature [8]. This conflicts with the general consensus that all regionally endothermic sharks are high trophic level macropredators, and that evolutionary pathways to gigantism in sharks (such as for *O. megalodon*) were facilitated by *either* regional endothermy *or* filter-feeding [10]; in *C. maximus* it appears *both* regional endothermy *and* filter-feeding may have played a role. In addition, gigantism itself reduces rates of specific heat transfer to the environment, however, gigantism alone does not confer steady-state elevated body temperature in the largest extant shark species which is a filter-feeder (*Rhincodon typus*; [20]; also see simulations in the supplementary material of [19]). Filter-feeding *C. maximus* were widely assumed to be fully ectothermic, as are several other species of Lamniformes [12,17]. Nevertheless, many extant members of the order are difficult to study given their biogeography, distribution and low abundance, which raises the possibility that regional endothermy is, and was, more prevalent in the evolutionary history of Lamniformes than previously thought. Indeed, it has been proposed based on fossil evidence that red muscle endothermy is an ancestral trait that evolved early in Lamniformes, approximately 113 Ma [10], but the point remains debated. In this study, we first present new data showing that *O. ferox*—an extant Lamniformes species with a fossil record that goes as far back as late Oligocene [21]—exhibits anatomical features characteristic of regionally endothermic sharks. We then consider this result with the recent discovery of regionally endothermic traits in basking sharks to propose a revision of the likely prevalence of red muscle endothermy in Lamniformes, and highlight several key implications of such a perspective.

## 2. Results and discussion

**Figure 1.**
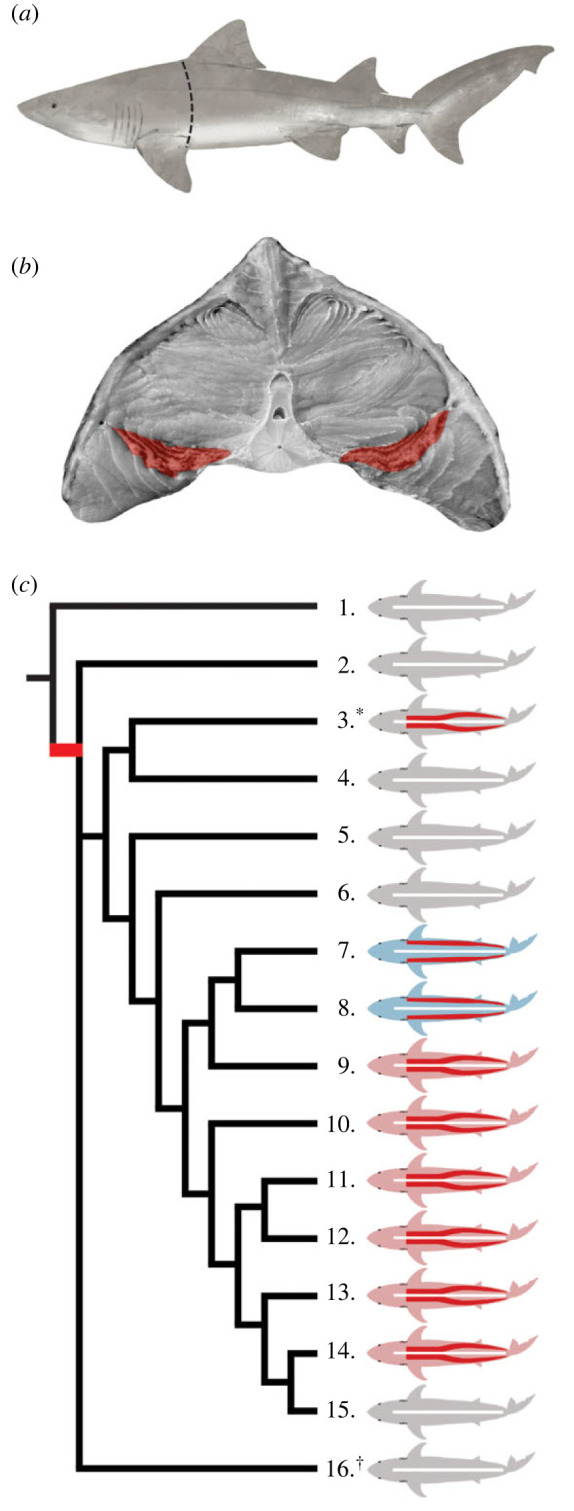
Red muscle distribution and prevalence of regional endothermy in Lamniformes. (*a*) Diagram of *O. ferox* showing location of transverse section indicated by black dashed line taken from specimen 1. (*b*) Posterior facing transverse section showing the medial to lateral band of centralized red muscle (red highlighted area) along the trunk, typical of regionally endothermic sharks. (*c*) Phylogram of Lamniformes adapted from Compagno 1990 [22] and Piemento *et al.* 2019 [10]. Extant species are presented, including *O. ferox, as well as the extinct *^†^O. megalodon*. Red muscle placement shown in species for which it has been documented (red bands within silhouettes; electronic supplementary material, table S1). Red muscle endothermy depicted by pink silhouettes, red muscle ectothermy depicted by blue silhouettes, and unconfirmed thermoregulatory strategy depicted by grey silhouettes. Proposed single origin of regional endothermy indicated by the red branch. Species depicted are numbered as followed: (1.) *Mitsukurina owstoni* (2.) *Carcharias taurus* (3.) *Odontaspis ferox (4.) *O. noronhai* (5.) *Pseudocarcharias kamoharai* (6.) *Megachasma pelagios* (7.) *Alopias superciliosus* (8.) *A. pelagicus* (9.) *A. vulpinus* (10.) *Cetorhinus maximus* (11.) *Lamna ditropis* (12.) *L. nasus* (13.) *Carcharodon carcharias* (14.) *Isurus oxyrinchus* (15.) *I. paucus* (16.) *^†^Otodus megalodon.* Red muscle endothermy in *C. maximus* assumed from measurements of sustained elevated subcutaneous white muscle temperature, but red muscle temperature and presence of vascular retia uncertain for this species.

Dissection of two stranded *O. ferox* specimens showed the species has centralized red muscle (a medial to lateral band along the trunk; figure 1*a,b*), an anatomical trait shared by all confirmed red muscle endotherm sharks examined to date [2,8,23]. Although the red muscle is not as centralized as in porbeagle *Lamna nasus* or salmon sharks *Lamna ditropis*, the red muscle extends closer to the vertebrae along the trunk than it does for red muscle ectotherms (such as blue sharks *Prionace glauca* and the pelagic and bigeye threshers *Alopias* spp*.* [2,24,25]), and is more similar to the distribution seen in red muscle endotherm lamnids and basking sharks [8]. The red muscle then becomes more laterally distributed from the second dorsal fin towards the posterior of the shark. Within the subcutaneous connective tissue and near the lateral extents of the red muscle, there appears to be a paired lateral artery and vein (figure 1*b*, electronic supplementary material, S1B). In confirmed red muscle endotherm Lamnidae species, a pair of subcutaneous vessels in a similar location then branch into heat conserving rete [26], although this has not yet been investigated in *O. ferox* (*or C. maximus*). The two *O. ferox* specimens we had access to did not lend themselves to histological analysis of the vasculature due to advancing tissue degradation. Analysis of the heart of one specimen showed *O. ferox* also has a high percentage of compact myocardium of the ventricle (48%); another trait shared among all regionally endothermic sharks examined to date. High proportions of compact myocardium is typically associated with elevated blood pressure and flow, potentially servicing the metabolic demands of regional endothermy (the potential link between regional endothermy and percent compact myocardium is discussed in detail in [8]). While we could not assess regional endothermy by taking *in vivo* temperature measurements of our carcasses, all studied shark species with centralized red muscle along the trunk are red muscle endotherms, with red muscle placement—in sharks at least—considered a ‘strong predictor’ of red muscle endothermy ([9]; but see, [27,28] for counter examples from teleosts).

If red muscle endothermy is found in *O. ferox*, then of the 15 extant species of Lamniformes, seven have red muscle endothermy, two are red muscle ectotherms (but might have cranial and orbital endothermy; [9]), and the thermophysiology of the remaining six are unknown (figure 1*c*). Accordingly, suspected red muscle endothermy in *C. maximus* and *O. ferox* suggests that this thermophysiology is more prevalent in Lamniformes than previously thought, particularly given earlier classifications of ectothermy are based partly on assumed links with feeding ecology that we now know to be tenuous (since the addition of filter-feeding *C. maximus* and deep-water benthivorous *O. ferox* add to the diversity of foraging modes). Consequently, it is possible that the remaining six extant Lamniformes, that have an unconfirmed thermoregulatory strategy, also exhibit features consistent with of regional endothermy. Dissecting further specimens in this group for red muscle placement, the presence of vascular retia, and biologging of body temperature would be informative.

These new possibilities for the prevalence of regional endothermy help reconcile some conflicting visions about evolutionary pathways to regional endothermy in Lamniformes, such as why *C. maximus* clusters closely with regionally endothermic sharks based on morphology [12], and whether regional endothermy evolved once ancestrally, or multiple times convergently, within the Lamniformes [9,10,12]. With the higher general prevalence and indications that *O. ferox* possibly has red muscle endothermy, our data support the single origin of regional endothermy in Lamniformes and that *O. megalodon* also likely possessed red muscle endothermy, and that pelagic and bigeye threshers subsequently lost it. The findings also raise questions regarding the role of regional endothermy in the development of gigantism and filter-feeding in sharks and rays, because it has been suggested that regional endothermy and filter-feeding are two mutually exclusive attributes that evolved to facilitate extreme body size, but *C. maximus* seemingly developed filter-feeding while retaining red muscle endothermy [8,10] and *Mobula tarapacana* is a large filter feeding ray with anatomical specializations consistent with red muscle endothermy [29].

Vulnerabilities that gigantism and regional endothermy likely impose on species given the increased whole-body energy requirements of both features are currently debated, particularly extinction risk under changing oceanic conditions [16]. Previous work suggests regional endothermy tends to be associated with higher extinction risk [19], and the much-debated cause of *O. megalodon* demise in the early Pliocene often focuses on high energetic demands due to regional endothermy coupled with changes in prey landscapes [14,16,19,30]. In this context, it is noteworthy that we now have evidence of possible red muscle endothermy in several extant Lamniformes with prey specialization at rather different trophic levels than previously recognized, particularly since *C. maximus* are gigantic (up to 12 m TL). It is therefore possible that filter-feeding is a critical adaptation that facilitates the persistence of gigantism, even during times of large biotic and environmental change. Indeed, it has been proposed that filter-feeding confers greater resilience to gigantic species than does regional endothermy, because of the higher abundance of small plankton compared to large prey [10]. Collectively, studies linking the appearance of regional endothermy to environmental change suggest that the evolution of regional endothermy took place during a time of low sea temperatures in the late Jurassic and early Cretaceous [31,32], which along with the subsequent evolution of gigantism, conferred sharks the ability to hunt in colder waters while avoiding competition with contemporaneous, gigantic, planktivorous bony fishes [33,34]. So, while the possible occurrence of red muscle endothermy in *C. maximus* and possibly *O. ferox* improves our understanding of the prevalence of regional endothermy and associated evolutionary pathways in sharks, it also contributes to our understanding of the role of different thermal strategies for extinction risk of elasmobranchs in warming oceans. This is important given most Lamniformes are severely vulnerable.

## 3. Methods

One carcass of a female *O. ferox*, measuring 433 cm total length (TL), was stranded on the east coast of Ireland in 2023 (specimen 1). Due to logistics of beach dissections, four transverse cross sections of the body were made just anterior of the first dorsal fin, anterior of the second dorsal fin, and anterior of the caudal peduncle to investigate red muscle distribution (electronic supplementary material, figure S1A, B). The heart was removed on site, rinsed with sea water, congealed blood was massaged from the organ [35], and then it was stored in a –20°C freezer for 2 days before thawing overnight to allow for dissection in the laboratory. Because the heart was large, the compact and spongey myocardium of the ventricle were dissected with a scalpel and forceps, then weighed on a scale (Brabantia International; Netherlands, 1 g accuracy). A second *O. ferox*, this time a male measuring 293 cm TL (specimen 2), was found floating at the surface by the public, who retrieved the body and kept it refrigerated (3–4°C) until collection and dissection 4 days later. This individual was gutted and sectioned into 11 full transverse cross sections of the body between the 4th gill slit and the precaudal pit (electronic supplementary material, figure S1A, C). See [36] for full details of stranded specimens.

A modified phylogram from Compagno [22] which matches recent molecular analysis at the genus level was reconstructed in Procreate software (version 5.2; Savage Interactive Pty Ltd) to include all extant Lamniformes and the extinct *Otodus megalodon* (figure 1*c*). Although there is no clear phylogenetic arrangement of extant Lamniformes and the extinct *O. megalodon* due to conflicting results from morphological and molecular analysis [10,12], multiple studies place *Mitsukurina owstoni* as the basal species, *C. maximus* as sister to Lamnidae, *Odontaspis* spp. being more basal to the more derived *C. maximus* and Lamnidae [37]. *O. megalodon* was included in this phylogram due to the interest in the origins of regional endothermy in the order and assumed life history. The placement of *O. megalodon* to extant Lamniformes, was based on analysis by Pimiento *et al*. [10], whereby the position of this species did not interrupt extant Lamniformes placement (as in Compagno [22]) as the interrelationships between this extinct species and the extant order is unresolved [10]. Anatomical and physiological features used to inform the modified phylogram are shown in electronic supplementary material, table S1.

## Data Availability

All raw data are contained within the manuscript file, with no additional data associated with the work. The data are provided in the electronic supplementary material [38].
